# Photocatalytic properties of Cu-containing ZnO nanoparticles and their antifungal activity against agriculture-pathogenic fungus

**DOI:** 10.1039/d2ra00863g

**Published:** 2022-03-29

**Authors:** F. Paraguay-Delgado, L. A. Hermida-Montero, J. E. Morales-Mendoza, Z. Durán-Barradas, Arturo I. Mtz-Enriquez, Nicolaza Pariona

**Affiliations:** Centro de Investigación en Materiales Avanzados SC (CIMAV), Laboratorio Nacional de Nanotecnología Miguel de Cervantes No. 120 31136 Chihuahua Chih México francisco.paraguay@cimav.edu.mx; Red de Manejo Biotecnológico de Recursos, Instituto de Ecología A. C Carretera Antigua a Coatepec 351, El Haya, 91073 Xalapa Veracruz México; Centro de Investigación y de Estudios Avanzados del IPN Unidad Saltillo Av. Industria Metalúrgica 1062, Parque Industrial Ramos Arizpe 25900 Coahuila México; Red de Estudios Moleculares Avanzados, Instituto de Ecología A.C Carretera Antigua a Coatepec 351, El Haya 91073 Xalapa Veracruz México nicolaza.pariona@inecol.mx conipariona@gmail.com

## Abstract

In this work, nanoparticles (NPs) of ZnO, ZnO with Cu incorporated at 2 and 30 wt%, and CuO were prepared by the hydrothermal method. X-ray diffraction pattern (DRX) analysis showed that ZnO with high Cu incorporation (30 wt%) generates the formation of a composite oxide (ZnO/CuO), while X-ray photoelectron spectroscopy (XPS) of the Cu (2 wt%) sample indicated that Cu is incorporated as a dopant (ZnO/Cu_2%_). The samples with Cu incorporated had enhanced visible light absorption. Methyl orange (MO) dye was used to perform photocatalytic tests under UV radiation. The antifungal activity of the NPs was tested against four agricultural phytopathogenic fungi: *Neofusicoccum arbuti*, *Alternaria alternata*, *Fusarium solani*, and *Colletotrichum gloeosporioides*. The ZnO/Cu_2%_ nanoparticles showed adequate photocatalytic and high antifungal activity in comparison to pure oxides and the composite sample.

## Abstract 2

In this work, nanoparticles (NPs) of ZnO, ZnO with Cu incorporated at 2 and 30 wt%, and CuO were prepared by the hydrothermal method.

## Graphical abstract

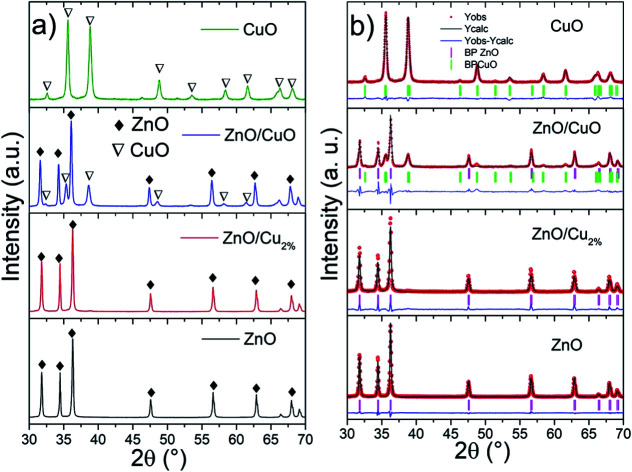

## 1. Introduction

Nanosized doped semiconductor oxides (SO) have shown improved biocidal and photocatalytic properties. They have been applied as antifungal agents,^1^ antibacterial agents,^2^ antivirals in agriculture,^3^ and photocatalysts for wastewater treatment.^4^ To improve these properties, SO are typically doped with transition metals (Mn, Fe, Cr, Cu),^5^ rare earth elements (Ce and Er),^6^ and noble metals (Au, Ag, Pt^7^). Another way to improve their antimicrobial and photocatalytic properties is by generating composite materials. One of the most used SO is ZnO, due to its attractive and unique characteristics such as wide bandgap, high exciton binding energy, low toxicity, variety of morphologies, and low cost.^8^ The tunable properties of ZnO are related to its morphology, particle size, surface area, and structural defects. Doped or composite SO are typically obtained by sol–gel, precipitation, and hydrothermal methods. The latter is adequate to obtain well-defined morphologies for particles with micro- and nanometric sizes. For several years, two-dimensional ZnO particles have been attracting attention due to their physical and chemical properties.^9^ The use of Cu as a dopant generates many changes in ZnO such as: (i) lattice distortion and morphological changes,^10^ (ii) improvement in photocatalytic activity in comparison with pure ZnO,^11^ (iii) increased energy conversion efficiency,^12^ (iv) improvements in the visible light absorption range,^13^ and (v) narrowing of the bandgap value.^14^ Cupric oxide is a widely used material in many applications due to its p-type character, narrow bandgap (1.2 eV), and visible light absorption.^15^ Many techniques are used to obtain CuO with different particle sizes and morphologies.^16^ Also, the incorporation of Cu^2+^ ions in excess into ZnO generates the formation of the CuO phase, giving ZnO/CuO composites. The formation of heterojunctions in ZnO–CuO has been shown to result in improved photocatalytic activity compared to those of pure ZnO or CuO.^17^ Additionally, SO nanoparticles have attracted interest as novel antifungal agents that can be applied to treat agricultural plants with high economic value.^18–25^

Wastewater treatment is a current concern due to its high potential to contaminate the environment. Additionally, it has a great economic impact, especially on developing countries.^26^ Processes in the textile industry consume very large amounts of water in and thus produce wastewater. Textile effluents may contain residues such as starch, waxes, NaOH, oils, metals, organic solvents, and dyes, depending on the process.^27^ Among all processes, the dyeing process has the greatest water consumption, meaning that a large amount of the produced wastewater is loaded with organic dyes.^28^ These dyes can be degraded into less-toxic or non-toxic compounds using advanced oxidation processes^28^ such as photocatalytic degradation. Additionally, fungal infections of crops are one of the main problems in agriculture.^29^

Phytopathogenic fungi have caused significant reductions in crop production, and some have caused epidemics. For example, the potato late blight was caused by *Phytophthora infestans*; this fungus triggered the Irish Great Famine in the 1840s.^30^ Hardwood trees can be affected by pathogenic fungi, which can reduce crop yield. For example, *Fusarium* dieback, a vascular disease caused by *Fusarium* spp. and other associated fungal species, affects avocado trees and ornamental trees.^31,32^ Depending on the fungus and plant species, fungi can affect different parts of the plant, including roots, fruits, stems, and leaves. One species of phytopathogenic fungus is *Fusarium solani*, which is found in soil and can infect a wide variety of crops (such as tomato, cotton, and potatoes) and can be transmitted to humans as well.^33^ As a soil-borne pathogen, the main target of *F. solani* is the roots of the plant, from which it propagates systemically and causes the death of the plant. Likewise, *Alternaria alternata*,^34^*Neofusicoccum arbuti*,^35^ and *Colletotrichum gloeosporioides*^36^ are pathogenic fungi that affect various crops, for example, avocado trees. The used organic azole fungicides have lowered efficiency due to the development of antifungal resistance.^37^ Therefore, semiconductor oxide NPs could offer a novel alternative that makes antifungal resistance harder. ZnO and CuO have shown great antifungal activity against a variety of phytopathogenic fungi. Some studies have reported selectivity in the antifungal effect of NPs depending on their chemical composition,^38^ while others show improved effects using doped or composite materials. The photocatalytic activity of these materials adds an antimicrobial effect in addition to the intrinsic effects shown by the material. This work compares the photocatalytic properties and the antifungal activity of three NPs, namely, ZnO, copper-doped ZnO, and ZnO/CuO composites. Their photocatalytic degradation of MO was evaluated under ultraviolet (UV) radiation, and their antifungal activity was tested against *Neofosicocum arbuti*, *Alternaria alternata*, *Fusarium solani*, and *Colletotrichum gloeosporioides*.

## 2. Experimental

### 2.1. Synthesis

All chemicals were of analytical grade and were used without further purification. The NPs were prepared following a hydrothermal method.^1^ Zinc acetate dihydrate (Zn(CH_3_COO)_2_·2H_2_O) and copper acetate monohydrate (Cu(CH_3_COO)_2_·H_2_O) were used as the zinc and copper precursors, respectively. First, 1.09 g of different proportions of the Cu : Zn precursor salts (0 : 100, 2 : 80, 70 : 30, and 100 : 0 wt%) and potassium hydroxide (0.84 g) were dissolved in 15 mL of tri-distilled water. Then, under constant stirring, 15 mL of ethanol (EtOH) was added dropwise at room temperature. Next, the resulting solution was transferred to a Teflon vial and placed inside a stainless-steel autoclave for heat treatment at 160 °C for 16 h. After this time, the NPs were washed three times with distilled water and dried at 90 °C for 4 h. The obtained NPs were named ZnO, ZnO/Cu_2%_, ZnO/Cu, and CuO based on the proportion of Cu : Zn precursor salts (0 : 100, 2 : 80, 70 : 30, and 100 : 0 wt%), respectively.

### 2.2. Characterization

The structural characterization of synthesized samples was done using XRD. The patterns were acquired using a PANalytical X'pert PRO diffractometer (Cu Kα (*λ* = 0.15406 nm)) in the Bragg–Brentano (*θ*–2*θ*) configuration; data was collected between 30° and 70° with a step size of 0.03° s^−1^. The patterns were refined using Thompson–Cox–Hastings for the samples, but in the case of the composite sample, a pseudo-Voigt function shape was used for the intensities; the Fullprof Suite program^39^ was used for Rietveld refinement. The particle morphology was studied using scanning electron microscopy (SEM). The images were acquired at different magnifications, operating at 5 kV and collecting electrons using a low electron image (LEI) detector. The microstructure characterization and elemental composition mapping images of the samples were obtained using a Hitachi 7700 TEM microscope. The particles were dispersed in ethanol by sonication for 5 min, and one drop of this suspension was placed on a 300-mesh lacey carbon-coated copper grid to study the ZnO sample, while a lacy carbon-coated nickel grid was used for the samples containing Cu. The absorbance spectra were acquired using a PerkinElmer Lambda10 spectrometer in the range between 200 and 1000 nm. Tauc plot methodology was used to determine the bandgap energy from the diffuse reflectance spectra using the Kubelka–Munk theory. The bandgap value was obtained by extrapolating the linear zone to the energy axis using the procedure outlined by Makuła *et al.*^40^ X-ray photoelectron spectroscopy (XPS) was carried out to determine the elemental composition and valence states of elements. An XSAM-HS KRATOS spectrometer was used and calibrated with the C 1s peak (BE = 284.6 eV) as a reference. Survey scans were used to detect the elemental contents of all the samples. High-resolution XPS spectra were acquired to analyze the O 1s, Zn 2p, and Cu 2p regions. Core and Auger region analyses were made using CasaXPS software employing LF lineshape, U2 Tougaard background, and the Levenberg–Marquardt method.

### 2.3. Photocatalytic activity

The photocatalytic properties of the samples were evaluated through the photodegradation of MO. Firstly, we formed a homogeneous coating layer of NPs inside a 60 mm glass Petri dish by adding 2 mL of an NP suspension (50 mg), after which the water was evaporated at 60 °C. To measure the photocatalytic activity, 5 mL of MO dye dilution (10 mg L^−1^) was poured into each coated Petri dish and irradiated with a UV lamp (315–400 nm). All samples were placed at the same distance from each lamp. The degradation process was evaluated at fixed time intervals; each time, 3 mL of MO dye dilution was withdrawn from each Petri dish, and the absorbance was measured at wavelengths between 300 nm and 600 nm using an Evolution 220 Thermo spectrophotometer. After measuring the tested sample, it was returned to the Petri dish and the experiment was continued. Each sample was tested in duplicate and the degradation percentage was determined using the following equation:
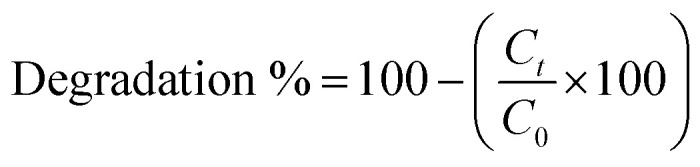
where *C*_*t*_ is the concentration of MO treated with NPs and *C*_0_ is the initial concentration of MO.

### 2.4. Antifungal activity

The antifungal activity was evaluated at three concentrations (0.5, 0.75, and 1 mg mL^−1^) against *Neofusicoccum arbuti* (strain INECOL_CBF-111), *Alternaria alternata* (strain INECOL_CBF-143), *Fusarium solani* (strain INECOL_CBF-171), and *Colletotrichum gloeosporioides* (strain INECOL_CBF-464) using the poisoned food technique. Those four phytopathogenic fungi were kindly provided by the laboratory of Biological Control of the Institute of Ecology A.C. Xalapa, Veracruz, Mexico. To determine the inhibition of the mycelial growth of the fungi, the fungal samples were incubated in potato dextrose agar (PDA) mixed with NPs. Mycelial plugs of 5 mm (cut from the periphery of 9-day-old fungus) were placed at the center of Petri plates containing different concentrations of NPs and then incubated for 9 days at 28 °C. There were four replicates for each treatment. After 9 days of incubation, the fungal growth area was measured, and the inhibition percentage was calculated with the following equation:
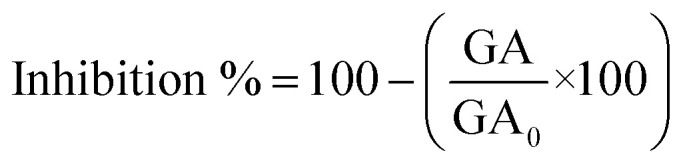
where GA is the growth area of the fungi treated with NPs and GA_0_ is the growth area of the control.

## 3. Results and discussion

### 3.1. Structure characterization

**Fig. 1:**
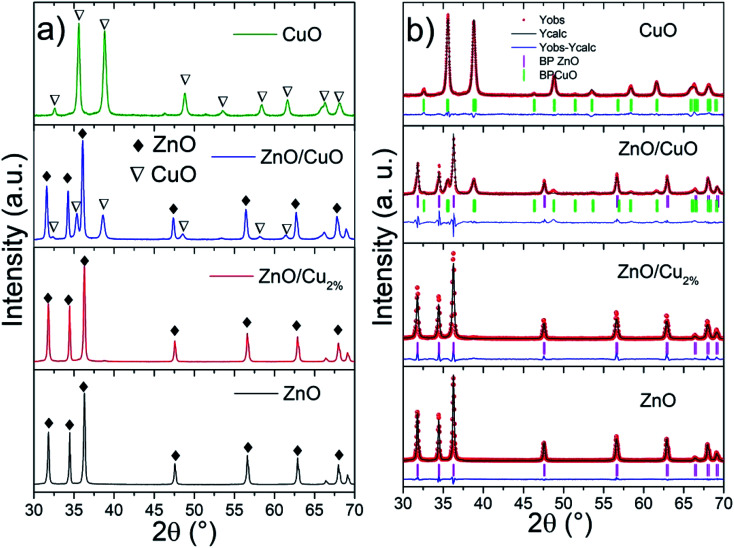
(a) XRD patterns and (b) Rietveld refinements for the ZnO, ZnO/Cu_2%_, ZnO/CuO, and CuO NPs.

The XRD patterns and Rietveld refinements of the samples are shown in Fig. 1. The patterns present good crystallinity with sharp intensity. The ZnO and ZnO/Cu_2%_ NPs patterns were indexed to JCPDS card no. 00-036-1451 (wurtzite-type hexagonal system for ZnO). The XRD pattern of the ZnO/CuO NPs was indexed to ZnO and CuO (JCPDS card no. 00-048-1548). The Rietveld refined method determined contents of 60 and 40 wt% for ZnO and CuO, respectively (Table 1). In the case of the CuO NPs, the XRD pattern was indexed to JCPDS card no. 00-048-1548. Fig. 1b shows the refined patterns for the NPs. The cell parameters for the ZnO/Cu_2%_ NPs are smaller than those of the pure ZnO NPs, which indicates a distortion of the ZnO cell due to the substitution of Zn atoms by Cu atoms. The refined cell parameters of the samples are reported in Table 1.

**Table 1:** XRD Rietveld refinement results, including the refinement precision value *χ*^2^, phases, and unit cell parameter values (Å)

Sample	*χ* ^2^	Phase %		ZnO (hexagonal)		CuO (monoclinic)			Cell vol.	
		ZnO	CuO	*a* = *b*	*c*	*a*	*b*	*c*	ZnO	CuO
ZnO	2.9	100	0	3.251	5.208				47.7	
ZnO/Cu_2%_	6.9	100	0	3.251	5.207				47.7	
ZnO/CuO	5.2	60	40	3.248	5.202	4.690	3.415	5.128	47.5	81
CuO	2.4	0	100			4.681	3.423	5.128		81

**Fig. 2:**
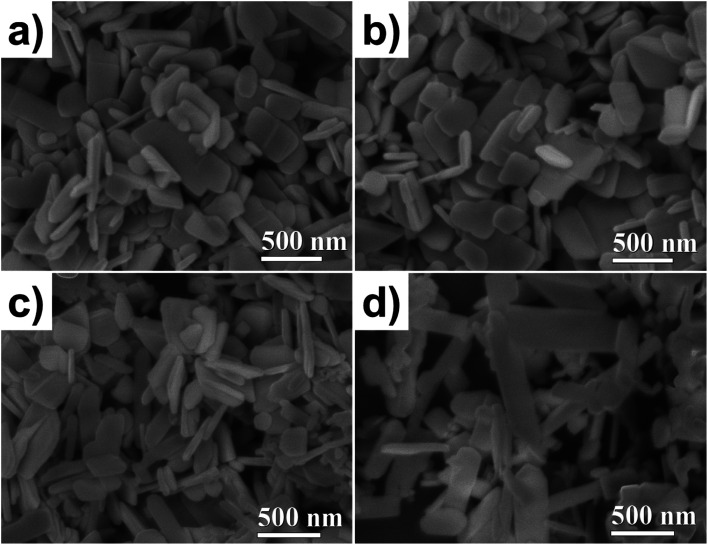
Particle morphology in SEM images for (a) ZnO, (b) ZnO/Cu_2%_, (c) ZnO/CuO, and (d) CuO NPs.

The particle morphology was studied using SEM. The images were acquired by secondary electrons at 40 kX magnification, see Fig. 2. The ZnO NP morphologies are shown in Fig. 2a; well-defined and homogenous lamellar particles can be seen with an average diameter and thickness of 300 and 50 nm, respectively. As shown in Fig. 2b, the doping did not modify the shape or size of the NPs. For the ZnO/CuO NPs, two kinds of particle morphology were observed (Fig. 2c), which means that two phases coexist (as revealed by XRD, Fig. 2a). The ZnO NPs exhibit a lamellar shape, but the CuO NPs have an elongated lamellar shape with 90° angles and different widths (Fig. 2d).

**Fig. 3:**
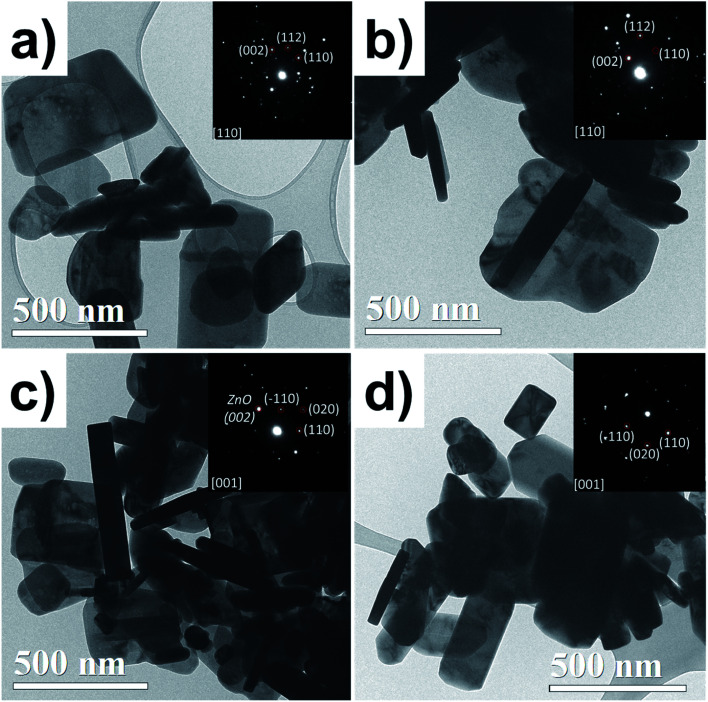
TEM bright-field micrographs for the (a) ZnO, (b) ZnO/Cu_2%_, (c) ZnO/CuO, and (d) CuO NPs. SAED patterns are inset for each.

Fig. 3 shows TEM bright-field images and SAED patterns. All samples show a flake-like shape; some of them are horizontal lamellar and others can be seen on the vertical side (darker ones), which could be noticed in the thickness of the particles. In the case of the ZnO NPs, the particle thickness is constant, and its SAED pattern (inset Fig. 3a) shows a crystalline particle, which was indexed to ZnO. The ZnO/Cu_2%_ NPs exhibited similar morphology to the ZnO ones, but with irregular contours attributed to the incorporation of the dopant Cu. The SAED from one particle shows a monocrystalline pattern indexed to ZnO. The coexistence of two types of particles was observed for the ZnO/CuO NPs; the ZnO NPs revealed a well-defined wider particle morphology, while CuO NPs had a more elongated lamellar shape, with clear parallelepipeds and rectangular edges, which helps us to easily identify them; the SAED pattern confirmed the CuO phase.

**Fig. 4:**
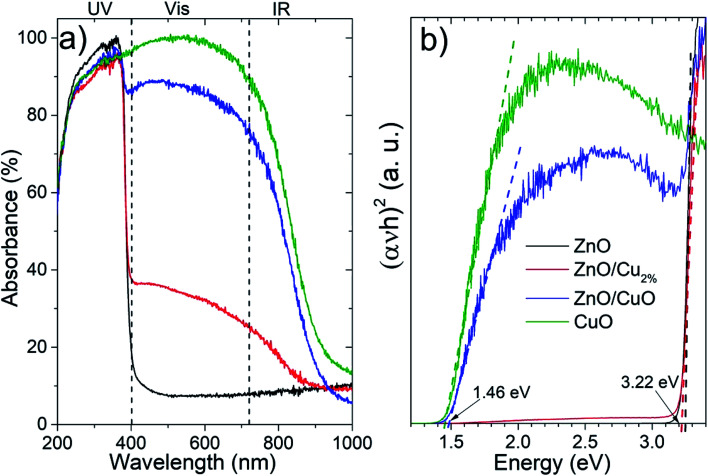
(a) Absorbance UV-vis-IR spectra and (b) bandgap determination using Tauc plots.

The absorbance spectra (Fig. 4a) of the NPs were acquired in the 200–1000 nm region. The ZnO sample shows a typical absorption in the ultraviolet region. There is a systematic increase in the absorption in the vis-region for the doped and composite samples. For the ZnO/Cu_2%_, NPs, the incorporation of a low percentage of the element Cu in ZnO generated an increase in absorption in the vis-region (∼30%), which was attributed to the creation of new interband energy states in the electronic band structure, while the ZnO/CuO NPs showed a high absorption of ∼85% in the vis-region. The CuO NPs showed strong absorption in the UV-vis range. Fig. 4b shows the Tauc plots used to determine the bandgap values; the bandgap for the ZnO and ZnO/Cu_2%_ NPs was 3.22 eV, indicating that the dopant does not generate modification. However, for the ZnO/CuO NPs, two band gaps can be distinguished, one corresponding to the ZnO (3.22 eV) and a second one corresponding to the CuO at 1.46 eV. As observed in Fig. 4b, the bandgap value for the CuO NPs was 1.46 eV.

**Fig. 5:**
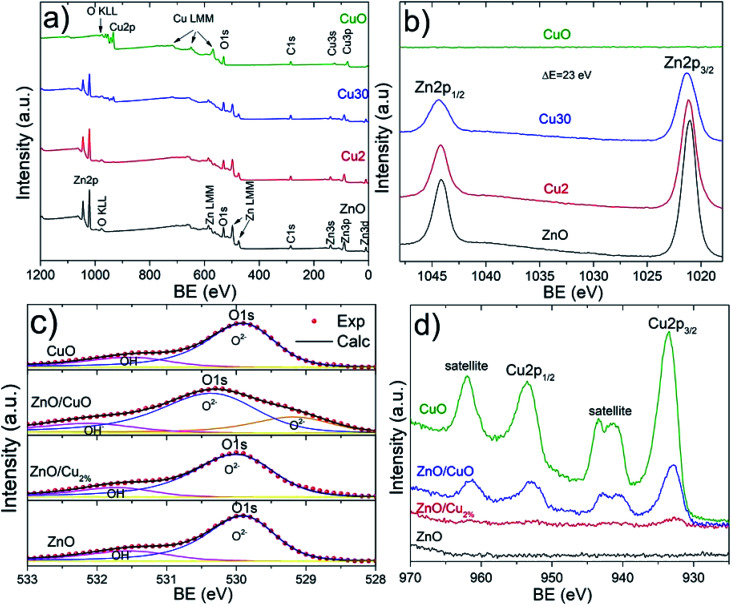
XPS spectra for all samples. (a) Survey spectra and (b) Zn 2p, (c) O 1s, and (d) Cu 2p regions.

The XPS spectra confirm the oxidation state of the studied samples. The survey spectra for the elements Zn, O, Cu, and C can be identified (Fig. 5a). Carbon peaks were used to calibrate each spectrum. Fig. 5b shows the high-resolution spectra of the Zn 2p region. Two characteristic edges were detected, Zn 2p_1/2_ located at 1044 eV and Zn 2p_3/2_ at 1021 eV, for the ZnO, ZnO/Cu_2%_, and ZnO/CuO NPs samples, corroborating the Zn^2+^ oxidation state. This valence state was determined from the binding energy difference between these edges, whose value is 23 eV for these three samples, confirming the formation of the ZnO phase. The O 1s region is shown in Fig. 5c; these spectra were deconvoluted using a pseudo-Voigt function. The ZnO, ZnO/Cu_2%_, and CuO NPs samples present behavior typical of metal oxides, with two components present. The first signal at low energy is attributed to Zn^2+^–O^2−^ bonding, and second at high energy is associated with the OH^−^ group on the surface of the material.^41^ In the case of the ZnO/CuO sample, there are three oxygen species: O^2−^, OH^−^, and the presence of additional components at lower energy (which is not clear). According to the literature, Cu exists in different cationic states forming the CuO phase or Zn–O–Cu compounds.^42^ The formation of the ZnO/CuO composite generates the presence of this component,^43^ which is associated with oxygen-deficient regions in the ZnCuO matrix.^44^Fig. 5d shows two component edges for the Cu 2p region, Cu 2p_3/2_, and Cu 2p_1/2_, which are located at 933.2 and 952.9 eV, respectively. The splitting value of these edges was approximately 20.2 eV for all samples, which is consistent with values reported in the literature. In the Cu 2p region two shakeup satellites are observed, which are located at 10.9 eV and 8.7 eV relative to the 2p_1/2_ and 2p_3/2_ edges, respectively. These shakeup satellites correspond to the CuO phase and the predominance of the Cu^2+^ oxidation state, which confirms the presence of this phase. The small intensity of the Cu 2p_3/2_ edge can be noted for the ZnO/Cu_2%_ sample, which is due to the presence of Cu as a dopant in the ZnO lattice, corroborating the incorporation of Cu^2+^ ions as a dopant.

The incorporation of Cu in the ZnO lattice generates a modification in the Zn 2p_3/2_ and Cu 2p_3/2_ edges. Table 2 shows the measurement values for both edges. Three parameters were compared: area, edge position (*X*_c_), and full width at half maximum (FWHM). The measured edge area provides information about how much Zn and Cu are in the samples. For example, in the case of the ZnO/Cu_2%_ and ZnO/CuO samples, the edge area for Zn 2p_3/2_ decreases compared to that in the ZnO sample; this variation is attributed to the presence of Cu as a dopant and the CuO phase, respectively. In addition, the Cu 2p_3/2_ edge area increases gradually for these NPs, showing a maximum value for the CuO samples. The edge positions in the ZnO and CuO samples indicate the elemental and chemical composition of NPs, showing the Zn^2+^ and Cu^2+^ oxidation states. The FWHM values are a useful indicator of the chemical state changes attributed to Cu incorporation. This broadening could show a relationship with doping and secondary phase formation for the ZnO/Cu_2%_ and ZnO/CuO samples, respectively. The FWHM of the Zn 2p_3/2_ edge presents a systematic increase with increasing Cu. The presence of the Cu 2p_3/2_ edge for the ZnO/Cu_2%_ sample corroborates the incorporation of Cu^2+^ ions in the ZnO lattice, while in the case of the ZnO/CuO sample, the presence of shake-up satellites confirms the formation of the CuO phase.

**Table 2:** High resolution XPS deconvolution data for the Zn 2p_3/2_ and Cu 2p_3/2_ edges

Sample	Zn 2p_3/2_ edge			Cu 2p_3/2_ edge		
	*X*_c_ (eV)	FWHM (eV)	Area	*X*_c_ (eV)	FWHM (eV)	Area
ZnO	1021.1	1.56	90 197	—	—	—
ZnO/Cu_2%_	1021.2	1.68	76 141	932.8	3.30	1909
ZnO/CuO	1021.3	1.97	57 671	933.1	3.00	12 976
CuO	—	—	—	933.6	2.95	40 277

### 3.2. Photocatalytic activity

**Fig. 6:**
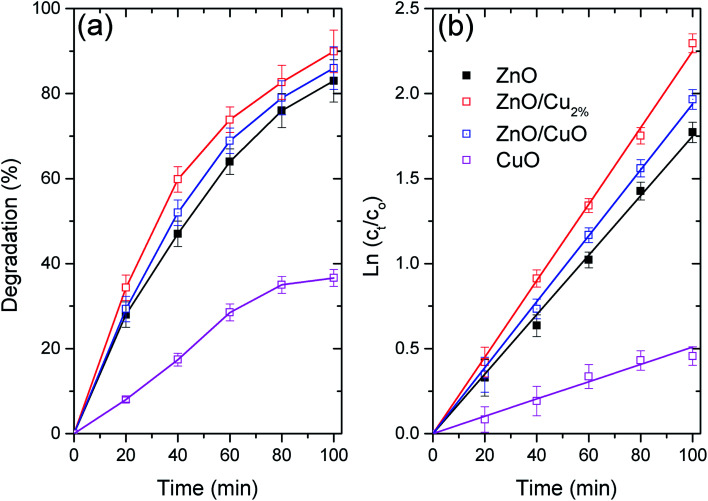
Photocatalytic degradation of MO with ZnO, ZnO/Cu_2%_, ZnO/CuO, and CuO under UV radiation. (a) Degradation percentages of MO, and (b) degradation data fitted using the first-order kinetic model.

Fig. 6a shows the photocatalytic activity of the three NPs as the degradation percentages of MO. Fig. 6b shows the degradation data fitted using first-order kinetics, for which linearized equation is: ln(*c*_*t*_) = ln(*c*_0_) − *k* × *t*, where *k* is the rate constant and *c*_*t*_ and *c*_0_ are the concentration of MO at time 0 and *t*, respectively. Table 3 shows the fitting parameters for the first-order kinetics model. The degradation data for the evaluated NPs follow first-order kinetics, which has been used to describe the degradation of various pollutants by ZnO-based photocatalysts (see Table 3).^45^ The highest degradation was found for ZnO/Cu_2%_ NPs, with nearly 90% degradation of MO in 100 min. The high photocatalytic activity of the ZnO/Cu_2%_ NPs is attributable to the Cu dopant incorporated in ZnO, which widens the absorption band as shown in Fig. 4a. The poor photocatalytic activity of the CuO NPs can be attributed to the fast recombination of electrons and holes and the photocorrosion of the material.^46^ Despite the high content of CuO (40%) in the ZnO/CuO sample, its photocatalytic activity was not much different from that of pure ZnO. Due to the photocorrosion that CuO can suffer, it is expected that ZnO/CuO would also have poor photocatalytic activity. However, the presence of the ZnO/CuO heterojunction, which is a p–n heterojunction, can improve the photocatalytic activity compared to that of either pure oxide by slowing the recombination rate of the electron–hole pair.^46^ Additionally, ZnO can also provide protection against the photodegradation of CuO by simply absorbing some of the radiation, making CuO last longer as a photocatalyst. Lastly, the ZnO/Cu_2%_ NPs had the best photocatalytic activity. They also include defects such as oxygen vacancies,^47^ which suppress the recombination rate of electron–hole pairs.^48^

**Table 3:** Fitting parameters for the first-order kinetics model and the correlation coefficients (*R*^2^)

Sample	*k* × 10^−3^	*R* ^2^
ZnO	17.5	0.999
ZnO/Cu_2%_	22.5	0.999
ZnO/CuO	19.4	0.999
CuO	5	0.992

### 3.3. Antifungal activity

**Fig. 7:**
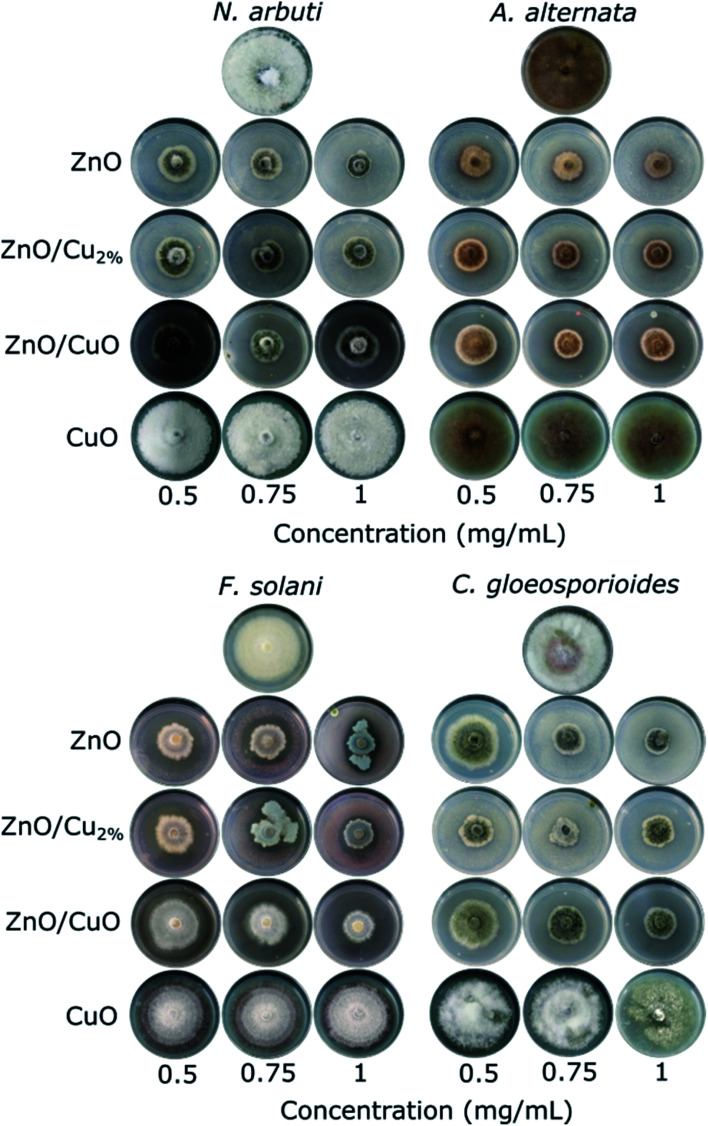
Antifungal activity of the ZnO, ZnO/Cu_2%_, ZnO/CuO and CuO NPs against *Neofusicoccum arbuti*, *Alternaria alternata*, *Fusarium solani*, and *Colletotrichum gloeosporioides*. Images in the same row correspond to specific NPs and columns indicate concentration. Untreated controls are at the top of each group.

The antifungal tests were evaluated by measuring the mycelial radial growth of four phytopathogenic fungi species. Fig. 7 shows the effect of the NPs on fungal growth; all the NPs affected the growth of the fungal colonies, except CuO NPs. It can also be observed that the antifungal activity of the NPs depends on the pathogenic fungi. Morphology changes were observed in the fungal colonies of *N. arbuti*, *F. solani*, and *C. gloeosporoides* treated with the three NPs (ZnO, ZnO/Cu_2%_, and ZnO/CuO NPs) (Fig. 7). Likewise, changes in the mycelia color from a lighter or white color to a darker one was observed. This could be due to the production of pigments generated by oxidative stress in the fungi in response to the NPs. On the other hand, the color changes from dark to light in the fungal colony of *A. alternata* were attributed to the synthesis of fungal pigments such as melanin, which can be found in this species.^49^

**Fig. 8:**
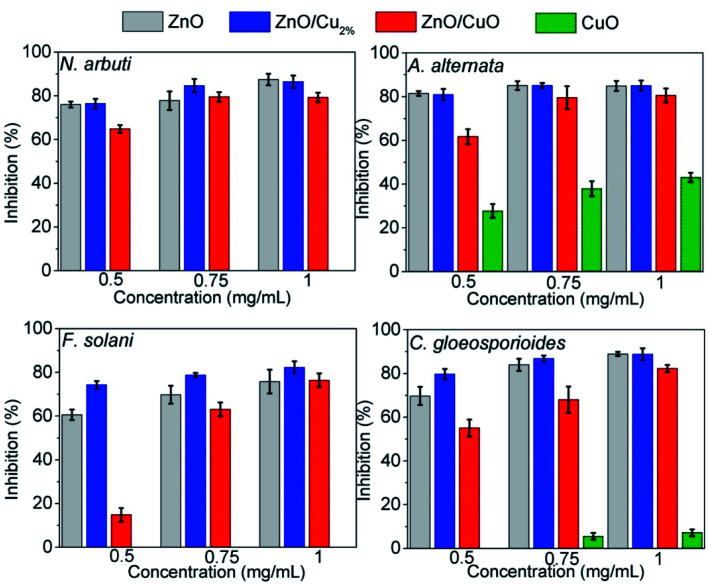
Inhibition percentages of ZnO, ZnO/Cu_2%_, ZnO/CuO and CuO against *Neofusicoccum arbuti*, *Alternaria alternata*, *Fusarium solani*, and *Colletotrichum gloeosporioides*.

Fig. 8 shows comparative bar graphs of the percent growth inhibition of the fungi for the different NP treatments. *A. alternata* and *C. gloeosporioides* were the most sensitive to ZnO/Cu_2%_ NPs; for concentrations as low as 0.5 mg mL^−1^, a high growth inhibition (>80%) was observed. The growth of *N. arbuti* and *F. solani* was inhibited 76.46% and 74.129%, respectively, by the ZnO/Cu_2%_ NPs. *A. alternata* (>80% growth inhibition) was the most sensitive to the ZnO NPs, followed by *N. arbuti*, *C. gloeosporioides*, and *F. solani* with 76%, 65%, and 60% growth inhibition, respectively. However, the percentages of growth inhibition are less than those for the fungi treated with ZnO/Cu_2%_ at the same concentration (0.5 mg mL^−1^). It can be pointed out that at high concentrations (0.75 and 1.0 mg mL^−1^) of the ZnO/Cu_2%_ and ZnO NPs, a slight increase in the percent growth inhibition of the four pathogenic fungi was observed. These results suggest that the used NPs have great antifungal activity and only 0.5 mg mL^−1^ is required to inhibit the growth of these fungi.

It was observed that the antifungal activity of the ZnO/CuO composite against four pathogenic fungi has a direct dependence on the concentration, meaning that the growth inhibition increased as the concentration increased (Fig. 8). The results show that *N. arbuti* and *A. alternata* are most sensitive to ZnO/CuO, but only 60% growth inhibition is achieved at 0.5 mg mL^−1^; unlike for ZnO, a higher concentration of ZnO/Cu_2%_ (0.75 mg mL^−1^) is required to inhibit 80% of the fungal growth. Likewise, to inhibit the growth of *F. solani* and *C. gloeosporioides* by >80%, 1 mg mL^−1^ of ZnO/CuO is needed, which means that these fungi are less sensitive to ZnO/CuO. The CuO NPs were the least effective antifungal, inhibiting *A. alternata* growth by only 40% at 0.1 mg mL^−1^. Furthermore, there was no growth inhibition for the species *N. arbuti* and *F. solani* using CuO NPs. Based on these results, the CuO phase reduces the antifungal activity of ZnO and the pure CuO phase shows less antifungal activity. However, when Cu^2+^ ions were incorporated into ZnO as a dopant, they increased the antifungal activity against the four pathogenic fungi. Thus, it can be inferred that oxygen vacancies are created due to the incorporation of Cu ions as a dopant, which modifies the electron states of the atoms (Fig. 5) and modifies the morphology of the particles (Fig. 3). The crystallographic defects generated at the ZnO NPs can help to produce more reactive oxygen species (ROS), such as ˙OH and ˙O_2_^−^, which are the main species produced by ZnO.^50,51^

## 4. Conclusions

In this work, the effect of Cu incorporation in ZnO NPs on the photocatalytic degradation of MO dye and antifungal activity against four fungal agricultural pathogens was studied. The pure oxide (ZnO and CuO), composite (ZnO/CuO) and doped (ZnO/Cu_2%_) NPs were synthesized by the hydrothermal method. The ZnO/Cu_2%_ NPs had the hexagonal ZnO phase with little lattice distortion generated by the incorporation of Cu, and elemental analysis confirmed the presence of the element Cu without drastic morphology changes. The ZnO/Cu_2%_ and ZnO/CuO NPs showed enhanced visible light absorption compared with pure ZnO, which is due to the formation of energy sub-levels in the electronic band structure for the doped sample and the presence of two phases for the composite sample. Additionally, the ZnO/Cu_2%_ NPs had higher photocatalytic activity compared to the other NPs. These activities were attributed to Cu doping, which changes or creates widening of the absorption band or leads to the generation of oxygen vacancies and slower recombination of the electron–hole pairs. Likewise, the ZnO/Cu_2%_ NPs were the most effective antifungal against four pathogenic agricultural fungi. Therefore, these results suggest these obtained materials with two-dimensional morphologies have great potential for remediation and are effective to control plant diseases caused by pathogenic fungi in agriculture and forestry.

## Conflicts of interest

There are no conflicts to declare.

## Supplementary Material
